# Long-term kidney function stabilization with fludrocortisone in autosomal recessive renal tubular dysgenesis: a case report

**DOI:** 10.1007/s00467-025-07051-2

**Published:** 2025-11-10

**Authors:** Reina Sugita, Shoichiro Kanda, Keiichi Takizawa, Yuko Kajiho, Yutaka Harita

**Affiliations:** The Department of Pediatrics, The University of Tokyo Hospital, 7-3-1 Hongo, Bunkyo-Ku, Tokyo, 113-8655 Japan

**Keywords:** Renal tubular dysgenesis, Fludrocortisone, Renin-angiotensin system

## Abstract

Renal tubular dysgenesis (RTD) is a rare disorder characterized by impaired development of the proximal tubules and kidney dysfunction due to decreased fetal renal plasma flow and renin-angiotensin system (RAS) inhibition. Fetal anuria causes severe oligohydramnios and Potter sequence, and most patients die within days after birth due to refractory hypotension, anuria, and respiratory distress. While vasopressin and fludrocortisone therapy have been reported, the long-term efficacy remains unclear due to the rarity of survivors. We report a 10-year-old girl with autosomal recessive renal tubular dysgenesis (ARRTD), a genetic form of RTD caused by mutations in RAS-related genes. She initially experienced recurrent dehydration, electrolyte abnormalities, and kidney dysfunction due to polyuria but showed long-term improvement following fludrocortisone therapy.

**Supplementary Information:**

The online version contains supplementary material available at 10.1007/s00467-025-07051-2.

## Introduction

Renal tubular dysgenesis (RTD) is a rare disorder characterized by impaired development of the proximal renal tubules, leading to kidney dysfunction [1]. It results from reduced renal plasma flow and suppression of the renin-angiotensin system (RAS) during fetal life. Fetal anuria occurs in most cases, leading to severe oligohydramnios and Potter sequence. Most affected neonates develop refractory hypotension and respiratory distress shortly after birth, often resulting in early death. Surviving cases are extremely rare, and even among survivors, chronic kidney disease often progresses due to recurrent dehydration and associated electrolyte abnormalities.

RTD can be classified into two forms: a primary form, autosomal recessive renal tubular dysgenesis (ARRTD), and secondary forms. ARRTD is caused by mutations in RAS-related genes such as *ACE*, *AGT*, *REN*, and *AGTR1*, resulting in impaired angiotensin II production and reduced secretion of downstream hormones including aldosterone and antidiuretic hormone (ADH). Secondary RTD has been described in infants with prenatal exposure to ACE inhibitors or angiotensin II receptor blockers, as well as in donor twins affected by twin-to-twin transfusion syndrome. Although ADH and fludrocortisone have shown therapeutic promise in ARRTD, surviving patients remain exceptional, and the long-term efficacy and optimal timing of such interventions have yet to be established.

Here, we report the long-term clinical course of a patient with genetically confirmed ARRTD. She initially presented with polyuria-induced dehydration, electrolyte abnormalities, and kidney dysfunction. Following fludrocortisone therapy, her clinical condition improved. We describe her course in detail and discuss the role of targeted hormonal therapy in ARRTD management.

## Case presentation

The patient is a 10-year-old girl born as the first child to parents who are not known to be consanguineous. She was delivered at 35 weeks and 5 days by emergency cesarean section due to severe oligohydramnios. Birth weight was 2336 g. No maternal use of ACE inhibitors or ARBs was reported. Soon after birth, she developed hypotension (BP 30 s/20 s mmHg) and respiratory distress, and underwent emergency surgery for idiopathic gastrointestinal perforation, followed by admission to the NICU. Postoperatively, she developed acute kidney injury, with persistent creatinine levels around 0.70 mg/dL, indicating chronic kidney disease (CKD). She also had bilateral joint contractures and a large anterior fontanelle. She was discharged at 1 month of age.

At 7 months, she was hospitalized for elevated creatinine (1.32 mg/dL) and hyperkalemia (6.1 mEq/L). Electrolyte abnormalities improved with hydration. A TTKG of 5.9 suggested type 4 renal tubular acidosis. Sodium chloride and sodium bicarbonate supplementation were started. Genetic testing at 14 months confirmed a homozygous *ACE* mutation (c.1454dup, p.Ser486Phefs*29), diagnosing ARRTD. This variant has not been previously reported in the literature.

At 2 years and 9 months, she was referred to our hospital for progressive CKD. Her estimated glomerular filtration rate (eGFR) was 48.23 mL/min/1.73 m^2^. Ultrasound showed increased echogenicity and poor corticomedullary differentiation without kidney enlargement; the longitudinal diameter of both kidneys was approximately 7.0 cm. Conservative treatment was continued.

At 3 years, she was hospitalized for acute kidney injury (AKI), hyponatremia, hyperkalemia, and non-anion gap metabolic acidosis after a cold. Laboratory tests showed a serum sodium level of 136 mEq/L, potassium 6.3 mEq/L, bicarbonate 18.8 mEq/L, and an eGFR of 31.2 mL/min/1.73 m^2^. Persistent polyuria, defined as a daily urine output exceeding 2000 mL, was observed during hospitalization (approximately 7.8 mL/kg/hr) and likely contributed to dehydration and pre-renal AKI. Although polyuria persisted, AKI and associated electrolyte abnormalities resolved with fluid therapy. Renin was markedly elevated at 18.5 ng/mL/hr (reference range: 0.2–3.9), while aldosterone was inappropriately low at 51 pg/mL despite being within the reference range of 36–240 pg/mL. These findings suggested impaired aldosterone response to renin stimulation. Serum ACE was markedly low at 0.9 U/L (reference range: 8.3–21.4), consistent with her underlying *ACE* gene mutation. In contrast, the plasma ADH level was 1.7 pg/mL (reference range: 0–2.8), indicating appropriate secretion in the context of volume depletion.

At 4 years, a similar episode occurred following another upper respiratory infection, with polyuria (approximately 6.6 mL/kg/hr) again leading to dehydration and AKI. After recovery, oral fludrocortisone (0.025 mg/day) was initiated.

**Fig. 1:**
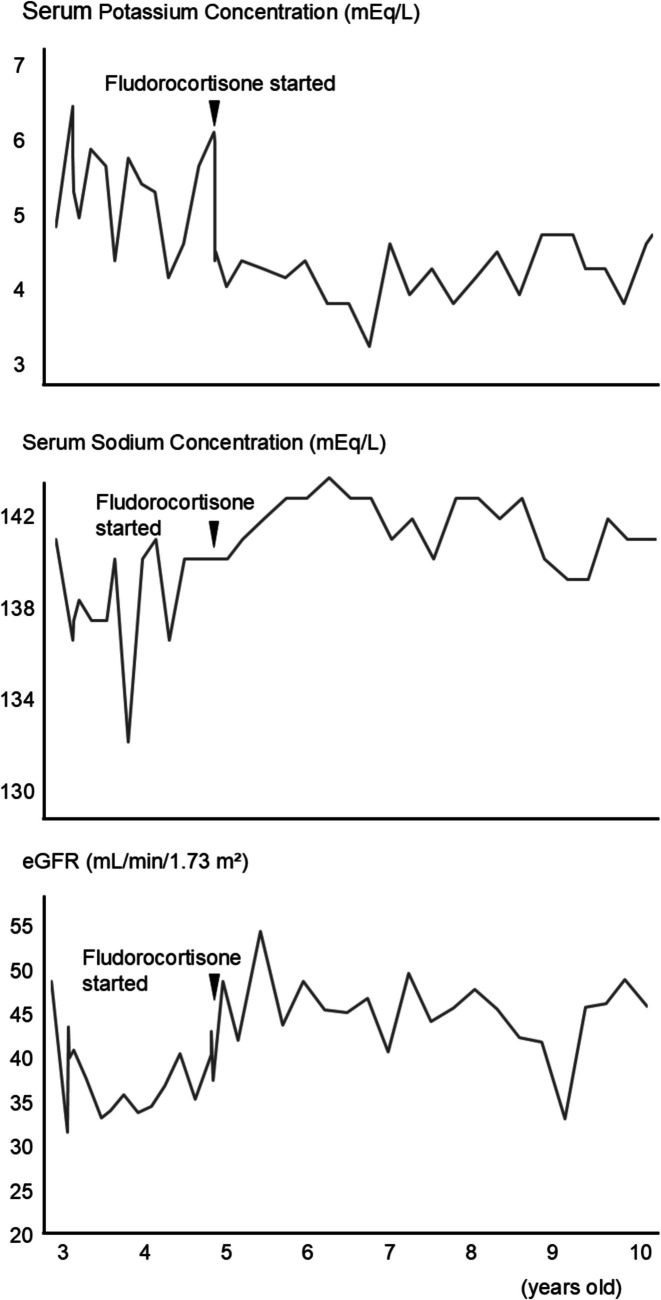
Changes in serum sodium levels, serum potassium levels and estimated glomerular filtration rate (eGFR) following the initiation of fludrocortisone therapy After starting fludrocortisone therapy, hyperkalemia and hyponatremia normalized, and eGFR improved over time

Following treatment, no further AKI or hyperkalemia episodes occurred (Fig. 1). Serum potassium levels, previously 5–6 mEq/L with frequent elevations, normalized. eGFR, which had been around 35 mL/min/1.73 m^2^, improved to around 45 mL/min/1.73 m^2^, and hyponatremia became infrequent. The patient and her family also noticed a reduction in daily urine output. Fractional excretion of sodium remained around 1.7–2.2% before and after fludrocortisone initiation, with no significant difference.

At age 10, her eGFR was 48.47 mL/min/1.73 m^2^, with stable kidney function. No further hospitalizations have been required. She is currently treated with sodium bicarbonate (3.6 g/day), sodium chloride (1.5 g/day), and fludrocortisone (0.04 mg/day). Her height and weight are within normal ranges, and neurodevelopment is age-appropriate.

## Discussion

This case describes a 10-year-old girl with ARRTD, who experienced recurrent episodes of hyperkalemia, metabolic acidosis, and pre-renal AKI, often triggered by dehydration. After initiating fludrocortisone therapy, these episodes resolved, and her kidney function stabilized.

ARRTD leads to impaired tubular development and typically presents in neonates with hypotension and respiratory distress due to oligohydramnios, often resulting in early death [1]. In this case, the patient was born after sudden-onset oligohydramnios and developed AKI and hypotension shortly after birth. Initial findings were attributed to septic shock and gastrointestinal perforation, but physical signs such as joint contractures and a large anterior fontanelle suggested underlying RTD. Previous reports have linked RTD with neonatal gastrointestinal perforation, possibly due to systemic hypoperfusion [2].

Endocrine abnormalities due to angiotensin II deficiency contribute to polyuria, electrolyte abnormalities, and volume depletion in ARRTD, which can lead to pre-renal AKI during dehydration. In our case, markedly elevated renin with relatively low aldosterone suggested insufficient aldosterone action, despite values within the normal range. The persistent hyperkalemia and acidosis implied that distal tubular immaturity reduced responsiveness to aldosterone.

Previous biopsy studies have shown preserved proximal tubular function but underdeveloped distal tubules in RTD kidneys [3], supporting this mechanism. The patient’s recurrent dehydration-induced AKI and electrolyte abnormalities were attributed to polyuria and impaired kidney hemodynamics. Similar findings have been reported in other RTD patients with high renin and low aldosterone levels [3]. Given the suspected aldosterone deficiency and distal tubular dysfunction, fludrocortisone—a synthetic mineralocorticoid—was administered.

Several case reports have demonstrated the efficacy of fludrocortisone in RTD, improving serum sodium and potassium levels [2, 4, 5]. In our case, fludrocortisone normalized potassium levels, reduced polyuria, and improved kidney function, with no further AKI episodes or hospitalizations. Notably, its effectiveness has also been reported even in early infancy, including in a 6-day-old [4] and a 1-month-old infant [2], and in improving hypotension in an 18-month-old [5]. These findings suggest that despite concerns about immature aldosterone responsiveness, mineralocorticoid therapy can be beneficial even in early life (Online Resource 1). Online Resource 2 summarizes the proposed pathophysiological mechanism of ARRTD and the therapeutic effect of fludrocortisone on kidney hemodynamics and sodium handling.

In summary, our case supports the use of fludrocortisone in ARRTD to correct electrolyte abnormalities and prevent recurrent AKI, thereby helping to stabilize kidney function over the long term.

## Summary

### What is new?

- Fludrocortisone therapy prevented recurrent AKI and electrolyte abnormalities in a patient with autosomal recessive renal tubular dysgenesis, leading to long-term stabilization of kidney function.

## Supplementary Information

Below is the link to the electronic supplementary material.

High Resolution Image (TIF 66.4 KB)

Online Resource 1 (XLSX 11.2 KB)

## Data Availability

Data sharing is not applicable to this article, as no datasets were generated or analyzed during the current study.
