# Supplementary figures and images for: Long-term kidney function stabilization with fludrocortisone in autosomal recessive renal tubular dysgenesis: a case report
Source: Pediatr Nephrol. 2025 Nov 10;41(3):679–82. doi: 10.1007/s00467-025-07051-2 (PMC12852240; doi:10.1007/s00467-025-07051-2)

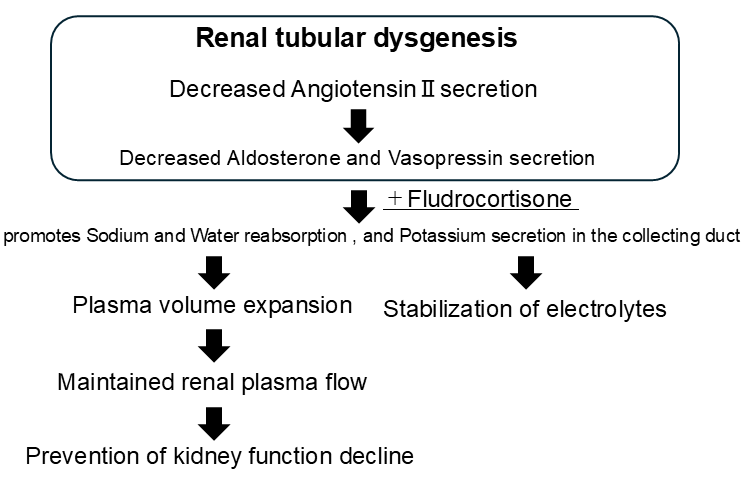

Supplement: Supplementary file 1 — High Resolution Image (TIF 66.4 KB) [file 467_2025_7051_MOESM1_ESM.tif]
